# Hermaphroditic freshwater mussel *Anodonta cygnea* does not have supranumerary open reading frames in the mitogenome

**DOI:** 10.1080/23802359.2017.1407705

**Published:** 2017-11-25

**Authors:** Marianna Soroka, Artur Burzyński

**Affiliations:** a Department of Genetics, Faculty of Biology, University of Szczecin, Szczecin, Poland;; b Department of Genetics and Marine Biotechnology, Institute of Oceanology Polish Academy of Sciences, Sopot, Poland

**Keywords:** Dui, mitogenomics, sex determination, orfans

## Abstract

The complete mitogenome of *Anodonta cygnea* is 15,613 bp long. This compact, circular molecule contains the set of 37 genes, typical for invertebrate mitogenomes, in the same order and orientation as in maternally inherited genomes of other bivalves from the same subfamily. There are only two unassigned regions longer than 200 bp (266 bp and 274 bp) and no indication of any supranumerary open reading frames.

*Anodonta cygnea* (Linnaeus, 1758) is a freshwater mussel from the family Unionidae, distributed in Eurasian waters. The family is several hundred species rich, but most of them are found in North America. They are usually gonochoristic, with the presence of two distinct mitochondrial lineages (M and F), inherited under DUI system (Skibinski et al. 1994; Zouros et al. 1994). This system has been faithfully operating in freshwater mussels for a long time, leading to extreme divergence of the two mitogenomes (Hoeh et al. 2002). Gender-specific anonymous open reading frames (FORF and MORF) have been described in both mitogenomes (Doucet-Beaupré et al. 2010). The few species with secondary hermaphroditism were described, and in case of North American mussels, these always lost the divergent, paternally inherited mitogenome. There were also substantial structural changes in the FORF (now denoted HORF) (Breton et al. 2011).

Here we announce, for the first time, the mitogenome of a European hermaphroditic species from the same family. We were unable to find a distinct paternally inherited mitogenome in sperm of this species so we assume the announced mitogenome to be the only one present.

The sample was taken in July 2009 from a pond in Hamrzysko village, central Poland. Identification down to species level was based on diagnostic morphological characters (Piechocki and Dyduch-Falniowska 1993). The specimen is stored under voucher number 328 in the local collection at University of Szczecin. The taxonomic identity was confirmed by comparison of the barcoding *cox1* sequence with the references (Bogan and Roe 2008).

The sequencing strategy followed the previously published three-step protocol (Soroka and Burzyński 2010). Two parts of the mitogenome were amplified with universal primers and sequenced. Species-specific long-range primers were used to amplify the rest of the mitogenome. The LR-PCR products were sequenced by primer walking. The complete mitogenome was assembled in *gap4* from Staden package (Staden et al. 2001). Annotations followed the established pipeline (Zbawicka et al. 2007) and were manually curated by comparison with the mitogenome of *A. anatina* (Soroka and Burzyński 2015).

The sequence has been deposited in GenBank under accession number MG385135. Comparative phylogenetic analysis was performed (Figure 1). The protein sequences encoded by the mitogenome differ from the closest relative (*A. anatina* F mitogenome) by approximately 10% (average p-distance, calculated in MEGA7 (Kumar et al. 2016)). No additional ORFs could be identified. In particular, the region containing FORF in *A. anatina* F mitogenome and HORF in *Utterbackia imbecillis* and *Lasmigona compressa* mitogenomes is much shorter and does not contain any ORF of appreciable length in *A. cygnea*.

**Figure 1.**
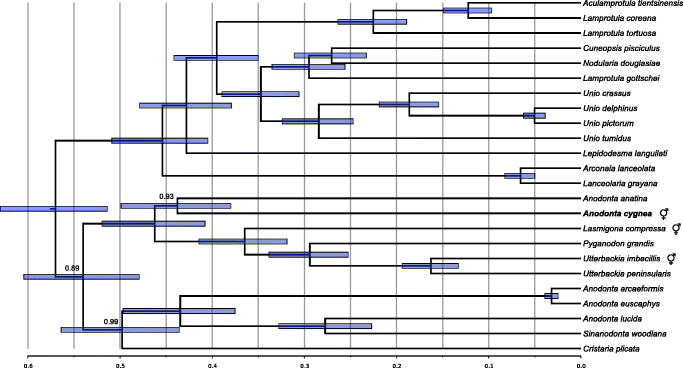
Phylogenetic tree showing the relationship of the announced mitogenome (in boldface) with the 23 closest relatives identified by BLAST search in *nr* database. It places *A. cygnea* as a sister taxon to *A. anatina.* The “latin name” fields of GenBank records were used to identify sequences, but several of these may be incorrect. In particular, the species related to *Sinanodonta woodiana* should most likely not be classified as *Anodonta*, likewise the three *Lamprotula* species should also follow different naming convention, most likely that presented recently by Lopes-Lima et al. (2017). The following records were used: *Aculamprotula tientsinensis* KR873102 (Wu et al. 2016), *Anodonta anatina* KF030964 (Soroka and Burzyński 2015), *Anodonta arcaeformis* KF667530 (An et al. 2016), *Anodonta euscaphys* KP187851, *Anodonta lucida* KF667529 (Song et al. 2016), *Sinanodonta woodiana* HQ283346 (Soroka 2010), *Arconaia lanceolata* KJ144818 (Wang et al. 2016a), *Cristaria plicata* GU944476 (Lee et al. 2012), *Cuneopsis pisciculus* KP273584, *Lamprotula coreana* JX050180, *Lamprotula gottschei* KJ018924 (He et al. 2016), *Lamprotula tortuosa* KC109779 (Wang et al. 2013), *Lanceolaria grayana* KJ495725, *Lasmigona compressa* HM856638 (Breton et al. 2011), *Lepidodesma languilati* KT381195 (Zhou et al. 2016), *Pyganodon grandis* FJ809754 (Breton et al. 2009), *Unio crassus* KY290446 (Burzyński et al. 2017), *Unio delphinus* KT326917 (Fonseca et al. 2016), *Nodularia douglasiae* KM657954 (Wang et al. 2016b), *Unio pictorum* HM014130 (Soroka and Burzyński 2010), *Unio tumidus* KY021078 (Soroka and Burzyński 2017), *Utterbackia imbecillis* HM856637 (Breton et al. 2011), and *Utterbackia peninsularis* HM856636 (Breton et al. 2011). Bayesian Inference, as implemented in BEAST (Bouckaert et al. 2014) was used to reconstruct the phylogeny. All the records were downloaded, reoriented to the common origin and aligned using ClustalW (Larkin et al. 2007). Since these genomes have the same structure and similar gene lengths, the only alignment ambiguities concerned the unassigned regions. However, these were inconsistent and have no influence on the final phylogeny due to complete elimination of columns with missing data. The optimal model of sequence evolution (GTR + G with relaxed, lognormal clock), matching the observed pattern of substitutions was selected, as previously described (Burzyński et al. 2017). The MCMC chains were run in quadruplicates for 20 × 10^6^ generations to reach ESS of at least 300 for each parameter. The four runs were convergent so the final tree samples were combined using *logcombiner.* The Maximum Clade Credibility tree was generated using *treeannotator*. The tree was visualized in FigTree (Rambaut 2009), and the root of the tree was scaled to match that of the recently published mitogenomic analysis (Burzyński et al. 2017). All nodes have posterior probabilities of 1.0, except for the ones indicated. The node bars represent 95% CI on node heights.

Of the three cases of secondary hermaphroditism covered by the presented data set, the *A. cygnea* case seems to be the only one without the HORF and also the oldest one (Figure 1; Mitchell et al. 2016). It can be concluded that after the loss of DUI, the supranumerary ORFs can eventually degenerate. This reinforces the hypothesis of the involvement of gender specific mitochondrial ORFs in sex determination of these animals (Breton et al. 2011).
